# Threat Diversity Will Erode Mammalian Phylogenetic Diversity in the Near Future

**DOI:** 10.1371/journal.pone.0046235

**Published:** 2012-09-28

**Authors:** Clémentine M. A. Jono, Sandrine Pavoine

**Affiliations:** 1 Museum national d’Histoire naturelle, Département Ecologie et Gestion de la Biodiversité, UMR CNRS UPMC 7204, Paris, France; 2 Mathematical Ecology Research Group, Department of Zoology, University of Oxford, Oxford, United Kingdom; University of Pretoria, South Africa

## Abstract

To reduce the accelerating rate of phylogenetic diversity loss, many studies have searched for mechanisms that could explain why certain species are at risk, whereas others are not. In particular, it has been demonstrated that species might be affected by both extrinsic threat factors as well as intrinsic biological traits that could render a species more sensitive to extinction; here, we focus on extrinsic factors. Recently, the International Union for Conservation of Nature developed a new classification of threat types, including climate change, urbanization, pollution, agriculture and aquaculture, and harvesting/hunting. We have used this new classification to analyze two main factors that could explain the expected future loss of mammalian phylogenetic diversity: 1. differences in the type of threats that affect mammals and 2. differences in the number of major threats that accumulate for a single species. Our results showed that Cetartiodactyla, Diprotodontia, Monotremata, Perissodactyla, Primates, and Proboscidea could lose a high proportion of their current phylogenetic diversity in the coming decades. In contrast, Chiroptera, Didelphimorphia, and Rodentia could lose less phylogenetic diversity than expected if extinctions were random. Some mammalian clades, including Marsupiala, Chiroptera, and a subclade of Primates, are affected by particular threat types, most likely due solely to their geographic locations and associations with particular habitats. However, regardless of the geography, habitat, and taxon considered, it is not the threat type, but the threat diversity that determines the extinction risk for species and clades. Thus, some mammals might be randomly located in areas subjected to a large diversity of threats; they might also accumulate detrimental traits that render them sensitive to different threats, which is a characteristic that could be associated with large body size. Any action reducing threat diversity is expected to have a significant impact on future mammalian phylogeny.

## Introduction

Current extinction rates are higher than would be expected from the fossil record [1]. In addition, if all of the species currently considered endangered disappeared during the next century, the extinction rate would be ten times higher than current rates [2]. To decrease this decline, researchers have searched for factors that threaten a species and for the causes of differences between similar species and their susceptibility to a given threat. Studies have also searched for potential consequences of species declines, especially in terms of phylogenetic diversity reductions, which is becoming a key criterion in conservation studies because it can reflect the variety of unique or rare features of a species [3].

In the current study, we focused on the expected loss of mammalian phylogenetic diversity. This class includes more than 5,000 species exhibiting an important diversity of lifestyles, and these species are charismatic and perform important ecosystem functions. Mammals are distributed throughout the world in a variety of habitats. However, their populations are quickly declining [4]: 25% of the mammal species for which there are adequate data available are at risk of extinction [5]. In 2008, the extinction risk status for mammals was updated in the Red List of the International Union for Conservation of Nature (IUCN) [6]; for most of the mammals listed, a phylogeny at the species level [7], [8] and biological data [9] are available. Consequently, many previous studies of species extinction risks have focused on mammals.

Previous studies have observed that phylogenetically related mammal species exhibit similar extinction risk levels and that species with few close relatives (in their genus, family and/or order) are more likely to be at risk [10], [11]. These two complementary trends could lead to a drastic loss of phylogenetic diversity if related species disappear [12]. Two explanations can be offered for this phylogenetic signal: 1) some species present a genetic predisposition toward extinction, and we expect some degree of correlation between the prevalence of extinction, phylogenetic position, and certain biological traits of a species - under this hypothesis, depauperate clades should have lost many species in the past because of their sensitivity to threats; and 2) species are subjected to local impacts, and closely related species exhibit a similar degree of extinction risk simply because they live in the same environmental conditions and experience a similar exposure to threats.

The trait-based explanation of extinction risk has been widely explored: groups of closely related species might share traits that influence their sensitivity to extinction risks (see Text S1). These traits have been used to forecast future extinction risk by identifying species that may present an intrinsic sensitivity to threats [12]. Species that are less at risk than predicted by these forecasting models might inhabit undisturbed regions or habitats, or they might cope relatively well in secondary disturbed areas. In contrast, species that are more at risk than predicted could be severely affected by extrinsic factors; for example, their habitat might simply be disappearing [13].

The level of extinction risk also depends on the threats to which species are exposed in the areas where they live. Indeed, threats vary worldwide depending on the environment, including the degree of human presence, cultures, and the practices of local people [14]. The prevalence of risk among mammals is higher in the Old World than in the New World and higher on islands than on continents [15], [16]. Australia has been affected by many recent species extinctions [17]. Threatened marine species are concentrated in the north Pacific and Atlantic, in southern Asia and in areas of high endemism [5]. These previous findings demonstrate that threats depend on where species live with respect to both geography and habitat.

Trait-based and geography-based explanations of species extinction risks are not necessarily exclusive. For example, extant species of phylogenetically old lineages of Bornean mammals are sensitive to timber harvesting and not able to cope with habitat change well. Most of these species are specialists (a potential trait-based cause of recent extinction risk, *e.g.*, [18], although this trait has not always been detrimental in the past [19]) and are endemic to insular Asia (a potential geographical cause of extinction risk [20]). In contrast, younger species are more tolerant, generalist, and geographically widespread, using all vegetation strata [21]. Biological traits, spatial position, and exposure to threats are critical elements in explaining and predicting losses of phylogenetic diversity.

The objective of the present study is to analyze the role of extrinsic factors in extinction risk. We mainly analyzed the effects of the type of threat and of the number of threat categories that affect a single species. We analyzed whether these effects could be explained by where species live in terms of their geographic location and associated habitats, with our findings indicating a prevalence of extrinsic factors over intrinsic factors of extinction risk. Finally, we highlight how the effects of intrinsic and extrinsic factors can integrate and affect the phylogenetic diversity of mammals. Our reasoning followed three sets of questions:

How much mammalian phylogenetic diversity is expected to be lost in the near future?
Is the threat type, as recently defined by the IUCN [22], likely to cause a bias in phylogenetic diversity losses because it specifically affects one clade in a phylogeny? Is this explained by geography and habitats?
Is threat diversity important in explaining the potential loss of phylogenetic diversity?

## Materials and Methods

### Extinction Risks, Threats, Geographical Areas, and Habitats

The IUCN Red List uses criteria including the decline in mature individuals, generation length, fluctuations in population sizes or distribution areas, and fragmentation into small populations to estimate species’ extinction risk [23]. We have used this list to classify each mammal species into one of the following categories [6]: Least Concern (LC), Nearly Threatened (NT), Vulnerable (VU), Endangered (EN), and Critically Endangered (CR). However, to prevent a potential bias in our analyses of extinction risk, we excluded species that are extinct or extinct in the wild and considered species for which limited biological information was available, or data-deficient species, in a separate analysis.

We transformed the IUCN categories of extinctions into numerical values of extinction probabilities, as defined by [24]. The model that was developed for this purpose, IUCN50, projects an IUCN extinction probability for the VU, EN, and CR categories at 50 years [23]. We use a short time range to reduce the uncertainties for future mammal species [24]. The extinction probabilities are as follows: LC = 0.00005, NT = 0.004, VU = 0.05, EN = 0.42, and CR = 0.97. We compared our results with two other models to verify that our main conclusions were not affected by the assessment of species’ extinction risk (Text S2).

For each species, we considered the major threat categories used by the IUCN (Table 1). Previous analyses of major threats that affect plants and animals were performed using a different threat classification system, which has been recently improved [22], [25]. For example, *Bison bonasus* (European Bison) was classified as endangered in the previous classification system, although no threats were identified for this species. Six distinct types of threats of the new classification system have now been included for this species on the Red List: urbanization, agriculture, transportation, biological resource use, human intrusion and disturbance, and disease. The old IUCN classification of threats has been changed partly because it was a conglomerate of stresses (*i.e.*, attributes of biodiversity targets impaired by human activities, such as reduced population sizes and habitat losses) and direct threats (*i.e.*, sources of stress = proximate human activities or processes that have caused, are causing, or may cause the destruction, degradation, and/or impairment of biodiversity targets) [22]. For example, an overriding threat, habitat loss, in the old classification system is now considered as a type of stress, and direct threats (*i.e.*, the sources of the stress) are now distinguished as agri- and aquaculture, urbanization, ecosystem change, and energy production (definitions in Table 1). Compared with the old classification system, the new system provides more detailed types of threats. For example, the natural disaster category is now differentiated into the effects of climate change and the effects of geological events [22].

**Table 1:** Short description of the IUCN classification of threats [6].

Threats	Abbreviation	Description and examples
1. Residential and commercial development	Urbanization	Human settlements or other non-agricultural land usages with a substantial footprint
2. Agriculture and aquaculture	Agri- & aquaculture	Farming and ranching as a result of agricultural expansion and intensification
3. Energy production and mining	Energy production	Production of non-biological resources (gas, mining)
4. Transportation and service corridors	Transportation	Creation of roads, railways, flight paths
5. Biological resource use	Harvesting/Hunting	Removal of individuals (*e.g.*, hunting)
6. Human intrusion and disturbance	Intrusion	Recreation, wars, military exercises
7. Natural system modifications	Ecosystem changes	Fire and fire suppression, dams
8. Invasive, other problematic species/genes	Exotics & pathogens	Non-native plants, animals, pathogens
9. Pollution	Pollution	Water-borne sewage, industrial pollution
10. Geological events	Geological events	Volcanic events, avalanches
11. Climate change and severe weather	Climate change	Aridity, storms, floods

Although the new classification system is improved, it still has limitations. Only direct threats are provided in the new classification system; however, direct threats (*e.g.*, pollution) and indirect threats (*e.g.*, the chemical factory that caused the pollution) may be difficult to distinguish. The next challenge to improve the analysis of threats is to develop a relevant classification for nested or interacting factors. The severity, in terms of expected population decline and scope, in terms of extension, of each threatening process are unknown for most taxa [22]. The relative role of a specific threat over other threats in determining the extinction risk of a certain species remains unevaluated on the IUCN Red List, the quantification of the severity and scope of each threatening process is a challenging task for future research [22].

The major threats defined by the new classification system are summarized in Table 1 [22]. Species currently classified as LC may experience threats if these threats decrease their population size, although their current population size has not yet reached the criteria of the IUCN risk classification system.

We also considered the geographical areas (Text S3) and the habitats (Table S1) defined by the IUCN Red List [6]. The geographical areas where each species lives were defined by the IUCN Red List based on GIS information for the countries where each species was found. Broad habitat categories, which take into account biogeography, depth in marine systems, and for inland aquatic habitats, the classification system of wetland types used by the Ramsar Convention, have been defined by the IUCN Red List. These habitat categories are in agreement with the definition of a habitat presented by Hall *et al.* [26], *i.e.*, the resources and conditions present in an area that result in the occupancy, including the survival and reproduction, of a given organism.

### Phylogeny

Mammalian phylogeny was defined by Bininda-Emonds *et al.* [7] and updated by Fritz *et al.* [8] to account for the more recent mammalian taxonomy of Wilson and Reeder [27]. This super-tree contained 92% of the mammal species with a known extinction risk and 75% of data-deficient species. Most of our analyses are performed at the species level, but we also highlight the main differences in extinction risk between the 23 monophyletic mammal orders.

### First Data Analysis: How Much Mammalian Phylogenetic Diversity are We Expecting to Lose in the Near Future?

For terrestrial mammals, if all of the species currently classified as VU, EN, and CR were driven to extinction, and all of the other species (including data-deficient species) remained, the amount of phylogenetic diversity lost would not be different from that expected if the VU, EN, and CR species were randomly distributed throughout the phylogeny [28]. Three extinction scenarios (including all NT and higher, all VU and higher, or all EN and higher of only Primates and Carnivores based on phylogenetic availability) showed that some mammal orders (Primates, but not Carnivores) could lose more phylogenetic diversity than randomly expected [11]. Phylogenetic diversity was measured in these previous studies using Faith’s PD index: the sum of branch lengths that connect species in a phylogenetic tree in terms of millions of years of evolution [3]. Here, instead of considering the extinction of all species at risk of extinction (VU, EN, and CR species), to answer the question “How much mammalian phylogenetic diversity are we expecting to lose in the near future?”, we used Faith’s index of expected future phylogenetic diversity [29], expPD: the sum for all branches of the phylogeny of the product of the length of the branch and the probability that all species descending from the branch are driven to extinction, assuming that extinctions are independent. This index uses estimates of a species’ extinction risk based on the IUCN50 model, where even LC species exhibit positive, although lower, probabilities of extinction, to predict the expected future phylogenetic diversity. We also benefited from the recent update of the mammalian phylogeny [7], [8] including all orders, both terrestrial and marine.

PD and expPD were used to analyze the relative expected loss of PD (PDloss), measured using the following equation: PDloss = (PD-expPD)/PD. Furthermore, we evaluated the hypothesis H0 stating that observed PDloss accurately represents what would be expected if the probabilities of extinction for all species were independent of the phylogeny. We performed our first test considering all mammals, followed by one test per order, as follows: 1) compute PDloss with the actual data; 2) permute (200 times) the extinction probabilities across all mammal species, and calculate PDloss based on permutated data; 3) calculate the p-value as the proportion of absolute PDloss values obtained via the permutational process that was higher than or equal to the absolute value of PDloss obtained with the actual data (two-tailed test).

To evaluate the impact of data-deficient species from these results, we repeated this analysis considering two extreme scenarios: (i) data-deficient species are all LC; and (ii) data-deficient species are all CR.

To interpret the results of these tests, we estimated the correlation between a mammal species’ distinctiveness and its extinction risk. The distinctiveness of a species is high if the species has few close relatives (*i.e.*, it descends from a long branch in the phylogenetic tree). In contrast, a species’ distinctiveness is low if it has many close relatives with recent common ancestors. We considered three indices of phylogenetic distinctiveness: ED [30]; ‘equal splits’, which we abbreviate as ES [31]; and an index based on ‘quadratic entropy’, which we abbreviate as QE [32]. We calculated the correlation between a mammal species’ distinctiveness and its extinction risk based on rank-transformed data in which the extinction risks are ranked in the following order: LC, NT, VU, EN, CR. We repeated this analysis by adding the data-deficient species as LC species and by adding them as CR species to evaluate the potential bias due to a lack of information regarding certain mammal species. Rank-transformed data were used (Spearman coefficient of correlation) for two reasons: (i) the relationship between a mammal species’ distinctiveness and estimates of its extinction risk is not expected to be linear; and (ii) extinction risk estimates are based on five discrete values with a distribution shape that is far from Gaussian. A linear relationship and a Gaussian distribution are required to use the parametric test based on Pearson correlation coefficient.

### Second Data Analysis: Is Threat type Likely to Cause Bias in Phylogenetic Diversity Loss?

The objective of the second data analysis was to determine whether a particular portion of the phylogeny was more affected by a particular threat. Using some of the threat types (mainly habitat loss, invasive species, and overexploitation) defined by the old classification scheme, it has previously been shown that different mammalian clades might be affected differently by different threat types (Table S2 in [33]). We complemented the aforementioned analysis by (i) utilizing all of the major threat types found in the new threat classification system (note that habitat loss is now considered as a stress instead of a source of stress [22]); (ii) analyzing data at the species level; and (iii) considering the phylogenetic distances among species. Thus, we analyzed whether species affected by a given threat type are clustered in the phylogenetic tree and the level at which they are clustered (*i.e.*, in broad clusters, such as orders, or fine clusters, such as small clades with recent common ancestor). To achieve this aim, we used a double principal coordinate analysis (DPCoA, [34]), which defines several axes to order threat types based on the phylogenetic positions of the species they affect. Only species affected by at least one threat were considered in the analysis. Definition of the pairwise phylogenetic distances between species was required for this analysis. We used the sum of the branch lengths along the shortest path that connected two species. DPCoA weighs each threat based on the number of species it affects to avoid the exaggerated effects of rare threats, such as geological events. DPCoA provides coordinates for the threats and coordinates for the species, which are then compared to determine which phylogenetic groups of species tend to be more affected by which threats.

Correspondence analyses [35] were used to determine the threat type distribution between geographical areas and habitats. A first correspondence analysis was applied to the table with threats as rows and geographic areas as columns to determine how many species are impacted by a given threat in a given geographic area. Correspondence analysis defines axes where threats and geographic areas are positioned. The coordinates of the threats and those of the geographic areas should be compared to determine the geographic areas that are dominated by various threats. We repeated the analysis twice, isolating land from marine geographic areas. A final correspondence analysis was applied to the table with threats as rows and habitat types as columns, which determined how many species are affected by a particular threat in a particular habitat. The correspondence analysis defines axes based on the position of threats and habitats. The coordinates of the threats and the habitats must be compared to determine the habitats dominated by certain threats.

### Third Data Analysis: Is Threat Diversity Important in Explaining the Expected Phylogenetic Diversity Loss?

First, we analyzed whether some of the threats (as defined in Table 1) are correlated in terms of the species they affect. Each threat was associated with a vector of 1 and 0, indicating which mammal species are affected by the threat. We next calculated the correlation between the occurrence vectors with the Phi coefficient (similar to the Pearson coefficient), which is insensitive to the number of species each threat affects [36] (Text S4).

Then, we measured the threat diversity with respect to the number of threats. We tested for phylogeny-, geography-, and habitat-based autocorrelations in the number of threats per species using Moran’s test ([37]; 999 permutations, see Text S5). We computed the correlation between the number of threats that affect each species and their category of extinction (both variables were rank transformed). We tested the significance of the correlation using permutation tests based on [38] to take into account the phylogenetic-, geography-, and habitat-based connections between species (see details in Text S5). All species, regardless of their extinction risk level, can be impacted by threats. For instance, 29% of the LC species are subjected to at least one major threat. There are various reasons for classifying these threatened species as LC. In some extreme situations, an LC species can be under a large number of threats. For example, *Sylvilagus bachmani* (Lagomorpha) has 13 recognized sub-species, each of which is affected by either a few threats or by no threats, and some of its subspecies maintain large population sizes in less perturbed areas, but because the threats are different from one sub-species population to another due to their different geographic locations, the species as a whole is affected by eight major threat types [6]. Conservation actions also maintain some species at an LC status, despite threat impacts. For example, *Mazama gouazoubira* (Cetartiodactyla) is impacted by seven threat types but is still classified as LC. The abundance of this species does not warrant a threatened status at this time because it is maintained in protected areas. However, populations are declining as they come into contact with human populations [6]. Moreover, all species, regardless of their extinction risk level, may not currently have identified threats. Species, classified as VU, EN, or CR, for which threats have not been identified may be poorly known species with as yet unidentified threats (*e.g.*, many Rodentia and Chiroptera species), species whose extinction risk is a result of a restricted geographic distribution, or species that experience fluctuations in population size due to varying stochastic threats (*e.g.*, insular CR species, *Peromyscus stephani*, Rodentia).

Consequently, to further evaluate the correlation between threat diversity and extinction risk, we tested the significance of the correlation by first removing all LC species and then removing all species without identified threats. We also analyzed whether threat diversity increases on average from LC to NT species, from NT to VU species, from VU to EN species and from EN to CR species using the exact test for comparison between means of discrete variables [39]. We performed this detailed analysis on all data, followed by removing all species without identified threats. Finally, we calculated the average number of threats that affect the species of each order and compared this value with the PDloss value using the Pearson correlation. Because some orders are far more affected than others, we verified that the rank-transformed data provided similar correlations. LC and non-threatened species were not removed from this analysis because PDloss (the expected future loss of phylogenetic diversity) depends on the positive probabilities of extinction for all species.

## Results

### Expected Phylogenetic Diversity Loss

With the IUCN50 model including only mammals for which adequate data are available, the observed mammalian phylogenetic diversity loss in 50 years (5.4%) is not quantitatively different from the loss expected if species’ extinction risks were independent of their phylogeny (average random loss = 5.8%, p = 0.100). When data-deficient species classified as LC are included in the model, the observed mammalian phylogenetic diversity loss in 50 years (4.7%) is also not quantitatively different from the loss expected if species’ extinction risks were independent of their phylogeny (average random loss = 4.9%, p = 0.135). In contrast, when data-deficient species classified as CR are added to the model, the observed mammalian phylogenetic diversity loss in 50 years (15.7%) is quantitatively higher compared to the loss expected if species’ extinction risks were independent of their phylogeny (average random loss = 14.6%, p = 0.005). Although the expected estimates of phylogenetic diversity losses depend on the model of species’ extinction probabilities (Text S2), the IUCN50 model is based on the actual designation of extinction risk categories determined by the IUCN Red List.

The phylogenetic diversity loss is expected to be higher if the most distinct species are the most threatened. However, we found low correlations between all indices of phylogenetic distinctiveness and the extinction risk: ED index, r = −0.038; ES index, r = −0.030; QE index, r = 0.086. The highest correlation is found for the QE index, which is more sensitive to distinctive clades, while ED and ES are more sensitive to distinct species (unpublished result), *i.e.*, to the branches closest to the tips in the phylogenetic tree [24]. This might confirm that distinctive clades with few species tend to be at higher risk [10], [11], but the correlation between the QE index and the extinction risk is too moderate to reach a conclusion on this issue. These correlations all decreased when considering data-deficient species as LC (ED index, r = −0.015; ES index, r = −0.006; QE index, r = 0.052) or CR (ED index, r = −0.021; ES index, r = −0.007; QE index, r = 0.035). Thus, the data-deficient species were not phylogenetically distinct.

Despite the lack of correlation between species’ distinctiveness and their extinction risk, we found that the expected future losses of phylogenetic diversity are not equivalent among orders. Not including data-deficient species, some orders could lose more PD than others, such as Perissodactyla, Primates, Cetartiodactyla, and Diprotodentia, whereas other orders could lose less PD, including Chiroptera and Rodentia (Table 2; see also Table S2 for other models of species’ extinction probabilities). Proboscidea and Monotremata could lose a high proportion of their phylogenetic diversity, although this could happen randomly without any phylogenetic signal in the species’ extinction risks, because of their low number of species and the shape of Monotremata tree, which includes four closely related and one distantly related species (*Ornithorhynchus anatinus*). When data-deficient species are classified as LC, the results are similar, whereas when data-deficient species are classified as CR, the following changes are observed: the proportion of missing data is higher in the orders with many LC species, which include those with a low expected PDloss; and when the data-deficient species are changed from LC to CR species, these orders become more threatened, and their expected PDloss is no longer lower than expected randomly. For one order, Cingulata, with many data-deficient species when converted to CR species, the expected PDloss is higher than what is randomly expected.

**Table 2:** Test that the hypothesis, H0 = observed loss in PD is not different from random loss (random extinction probabilities).

		Without data-deficient species		With data-deficient species as LC		With data-deficient species as CR	
Order	n	Relativeloss (%)	P-value	Relativeloss (%)	P-value	Relativeloss (%)	P-value
Hyracoidea	4(0)	0.005	0.370	0.005	0.165	0.005	0.180
Paucituberculata	5(0)	0.833	0.525	0.833	0.605	0.833	0.375
**Cingulata**	17(3)	0.935	0.200	6.412	0.220	**23.536**	**0.035 (+)**
**Didelphimorphia**	**68(14)**	**1.763**	**0.020 (−)**	**1.405**	**0.030 (−)**	18.024	0.470
Pilosa	9(0)	2.517	0.840	2.517	0.905	2.517	0.465
**Chiroptera**	**880(153)**	**3.179**	**0.005 (−)**	**2.611**	**0.005 (−)**	13.162	0.240
Afrosoricida	38(4)	3.209	0.390	2.963	0.405	10.404	0.370
Sirenia	4(0)	4.146	0.905	4.146	0.915	4.146	0.575
Macroscelidea	12(3)	4.271	0.870	3.991	0.945	10.321	0.770
Carnivora	252(19)	4.444	0.395	4.147	0.700	10.488	0.145
**Rodentia**	**1735(290)**	**4.813**	**0.010 (−)**	**4.101**	**0.010 (−)**	16.358	0.150
Scandentia	17(3)	5.020	0.610	4.607	0.785	12.600	0.540
Pholidota	8(0)	5.868	0.950	5.868	0.925	5.868	0.410
Eulipotyphla	330(61)	6.658	0.720	5.845	0.685	17.699	0.260
Dasyuromorphia	61(1)	6.819	0.710	6.662	0.460	8.435	0.070
Lagomorpha	81(5)	6.956	0.510	6.632	0.255	8.518	0.260
Peramelemorphia	16(2)	8.729	0.560	7.889	0.485	17.225	0.800
**Diprotodontia**	**122(2)**	**9.203**	**0.025 (+)**	**9.127**	**0.010 (+)**	9.935	0.200
**Cetartiodactyla**	**237(53)**	**10.061**	**0.005 (+)**	**7.436**	**0.035 (+)**	**29.747**	**0.005 (+)**
**Primates**	**304(15)**	**12.238**	**0.005 (+)**	**11.188**	**0.005 (+)**	15.925	0.080
Monotremata	4(0)	17.088	0.120	17.088	0.090	17.088	0.950
Proboscidea	2(0)	23.500	0.115	23.500	0.095	23.500	0.870
**Perissodactyla**	**14(0)**	**25.502**	**0.005 (+)**	**25.502**	**0.005 (+)**	**25.502**	**0.035 (+)**

We used the IUCN50 model of species’ extinction probabilities (see Table S2 for other models). The three orders with only one species in our dataset were removed from the table. The order Notoryctemorphia, with 2 data-deficient species, was also discarded. *n* = number of species that are not classified as data-deficient, and the number of data-deficient species is given in brackets; relative loss = PDloss. Significance tests with α = 5% are highlighted in bold; a sign in brackets indicates whether PDloss is higher (+) or lower (**−**) than expected randomly. Orders are presented in increasing order regarding PDloss when data-deficient species are discarded.

### Different Threats Affect Different Clades

#### Different types of threats affect distinct parts of the phylogeny

**Figure 1:**
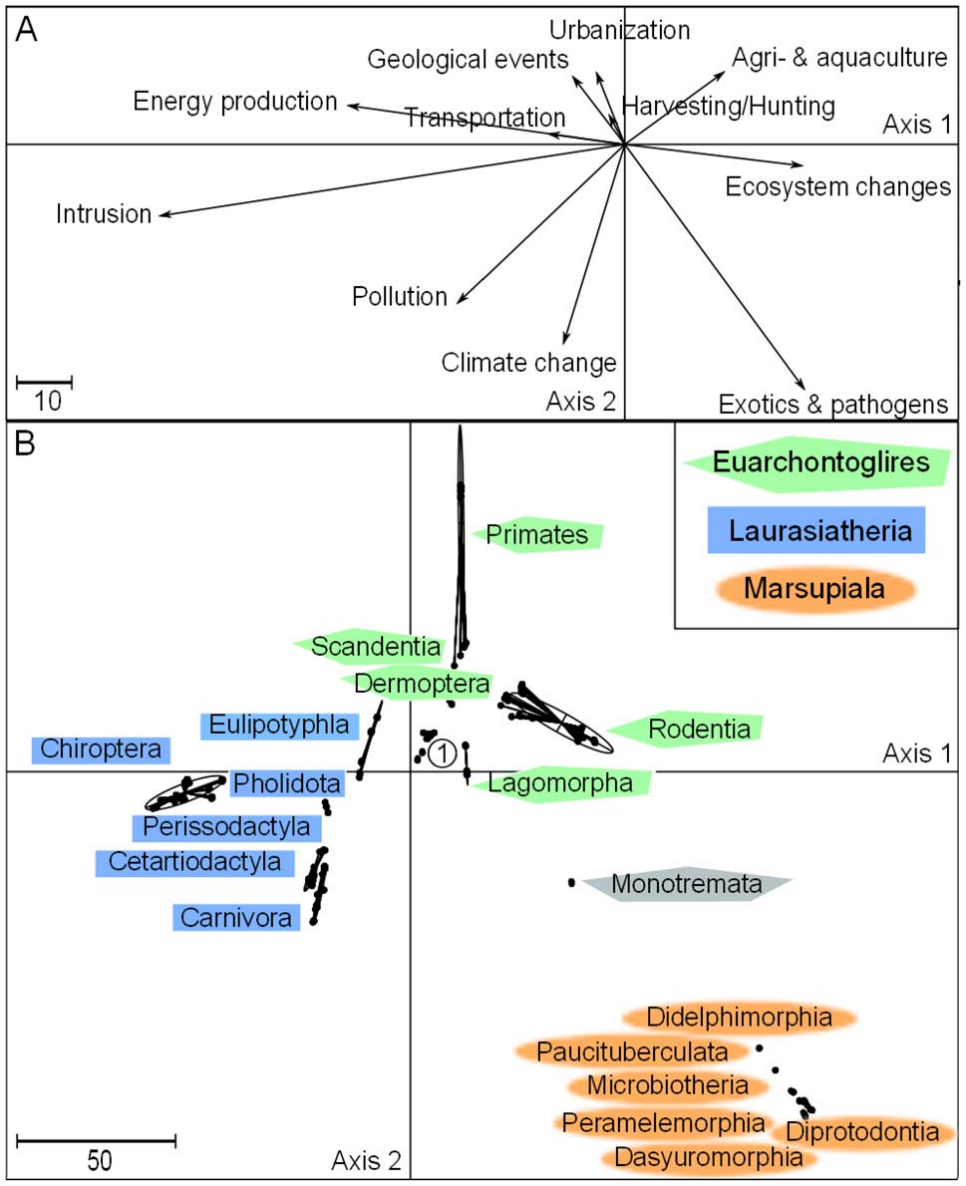
DPCoA axes describing which threats tend to affect which mammalian lineages. (A) Coordinates of the threats on each axis. (B) Coordinates of the species, grouped by orders and higher level clades. Extant mammals are grouped into two subclasses: Theria and Protheria. The only extant Protheria are Monotremata. Theria is divided into Eutheria (with Xenarthra, Afrotheria, Euarchontoglires, and Laurasiatheria) and Metatheria (with Marsupiala). The coordinates of the threats and the species must be associated to interpret the graphs: *e.g.*, the Marsupiala, located on the negative side of the second axis, tend to be more affected by threats that are also located on the negative side of the second axis, including exotics and pathogens. In panel (B), the encircled number 1 indicates the location of Xenarthra and Afrotheria.

The first two ordination axes of the DPCoA represented 52% and 22% of the phylogenetic differences between the species affected by different types of threats, respectively (Fig. 1). Marsupiala and Monotremata tend to be more affected by exotic species, climate change, and pollution than any of the other phylogenetic groups. Chiroptera are highly distinctive because they are primarily affected by human intrusion and disturbance, and to a lesser extent, they are affected by energy production and mining. Euarchontoglires are more affected by urbanization, agriculture, harvesting and hunting, especially among certain Primate families (Cebidae, Aotidae, Atelidae, Callitrichidae, Hominidae, Hylobatidae, Pitheciidae and Cercopithecidae). The other Primate families presented greater proportions of species affected by energy production, ecosystem change and pollution (threats shared with the other orders; see Table S3).

#### Connections between threat types, geography, and habitats

**Figure 2:**
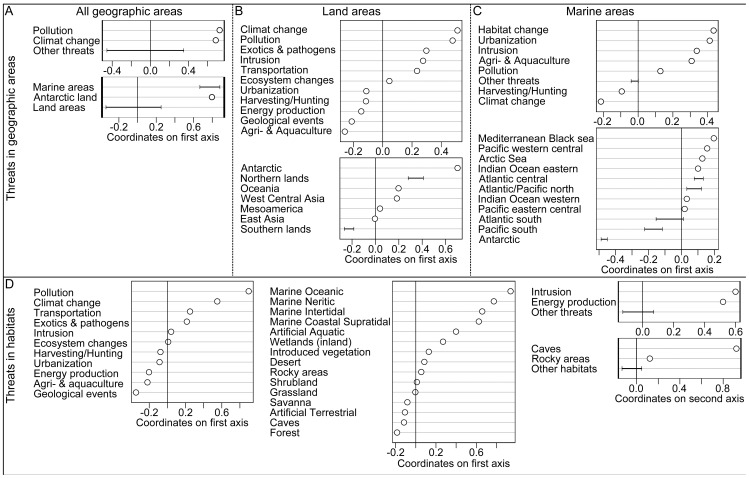
Correspondence analyses of the threats found among geographic areas and habitats. (A) All geographic areas (the first ordination axis expresses 69% of the association), (B) land areas (61%), (C) marine areas (45%), and (D) habitats (the first two axes represent 52% and 33% of the association). Threats and geographic areas (or habitats) must be associated for interpretation. For instance, in (A), pollution and climate change, located on the positive side of the first axis, tend to be associated with marine areas and Antarctic land, which is also located on the positive side of the first axis. To simplify the graphs, some geographic areas, habitats, and threats have been grouped, and the range of their coordinates is given by a segment.

Correspondence analysis first determined that marine areas were predominantly affected by pollution and climate change. Next, this analysis revealed differences between the threats that affect the Northern and Southern Hemispheres in both land and marine areas (Fig. 2A, B, C). For land areas, species living in the Northern Hemisphere are mostly threatened by climate change and pollution and, to a lesser extent, by exotics and pathogens, human intrusion and transportation. In contrast, species living in the Southern Hemisphere are mostly threatened by agriculture, energy production, harvesting/hunting and urbanization. The few species affected by geological events are primarily distributed in the Southern Hemisphere. Oceania and Antarctica are subjected to threats similar to those affecting the Northern Hemisphere (Fig. 2B). In marine areas, species in the Northern Hemisphere tend to be more threatened by changes in ecosystems, urbanization, human intrusion, and aquaculture. In contrast, species in the Southern Hemisphere were found to mainly be threatened by climate change. The last correspondence analysis, which examined the association between threats and habitat types, mainly contrasted terrestrial and marine habitats, as expected. The final correspondence analysis also revealed that the threats that affect freshwater habitats are similar to those affecting marine habitats (Fig. 2D). Finally, the cave habitat was distinguished from the other habitat types because it was mostly affected by human intrusion and disturbance, energy production, and mining (Fig. 2D).

### Threat Diversity Determines a Species’ Extinction Risk

All of the Phi correlations between threat types and the species they affect were lower than 0.25, except for the following six pair-wise correlations: φ(Agri- & Aquaculture, Harvesting/Hunting) = 0.58; φ(Urbanization, Agri- & Aquaculture) = 0.43; φ(Urbanization, Harvesting/Hunting) = 0.36; φ(Urbanization, Transportation) = 0.33; φ(Agri- & Aquaculture, Ecosystem changes) = 0.30; and φ(Transportation, Harvesting/Hunting) = 0.25.

**Figure 3:**
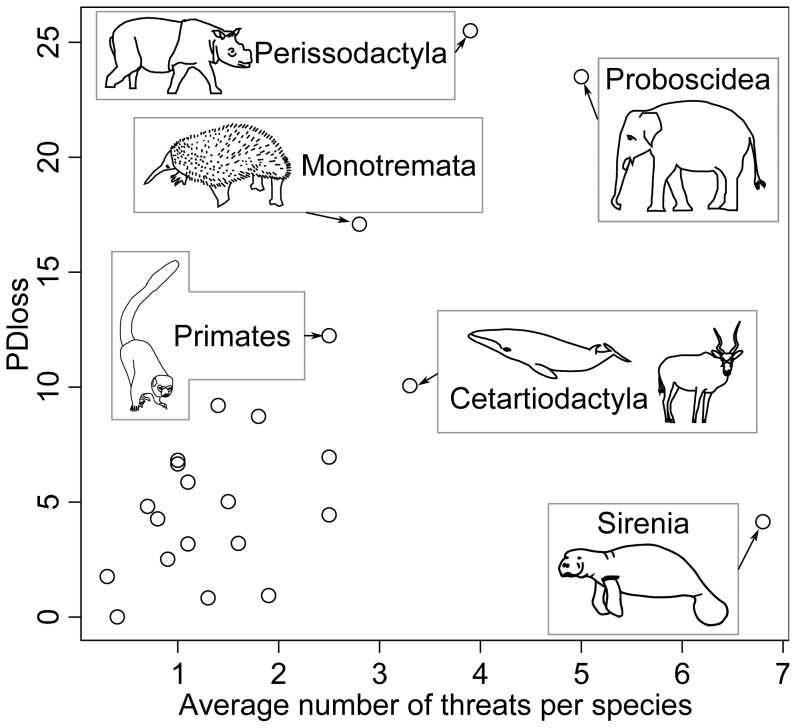
Expected relative loss of phylogenetic diversity explained by the average number of threats per species. We used the IUCN50 model of species’ extinction probabilities (see Fig. S1 for other models). The expected relative loss of phylogenetic diversity and the average number of threats per species were calculated for each mammal order. The names of the orders with the highest combined number of threats and expected relative PD loss have been indicated. The atypical position of the Sirenia is also indicated. Drawings of threatened species among these orders have been added, including *Dicerorhinus sumatrensis* (critically endangered Sumatran Rhinoceros, Perissodactyla), *Elephas maximus* (endangered Asian Elephant, Proboscidea), *Zaglossus bartoni* (critically endangered Eastern Long-beaked Echidna, Monotremata), *Eulemur Mongoz* (vulnerable Mongoose Lemur, Primates), *Addax nasomaculatus* (critically endangered Addax, Cetartiodactyla), and *Trichechus manatus* (vulnerable West Indian Manatee, Sirenia).

The low correlation between threats led us to estimate threat diversity as the number of threats that affect a single species. Phylogeny-, geography-, and habitat-based autocorrelations on the number of threats per species were all significant (Moran’s tests: n = 4223, I = 0.0151 p = 0.001, I = 0.0300 p = 0.001, I = 0.0098 p = 0.001, respectively). Correcting for phylogenetic-, geography-, and habitat-based connections between species, we found a significant correlation between the number of threats that impact a species and its level of extinction risk when all species were considered (r = 0.62, n = 4423, p = 0.005, with all corrections), as well as when LC species were removed (r = 0.10, n = 1282, p = 0.005 with all corrections), and when species without any identified threat were removed (r = 0.19, n = 2078, p = 0.005 with all corrections). When all species were included in the analysis, the average number of threat types per species increased when the level of extinction risk transitioned from LC to NT species (T = 20.99, p = 0.005 with all corrections) or from NT to VU species (T = 2.95, p = 0.005 with all corrections). However, the number of threats was not different when the level of species extinction risk transitioned from VU to EN species (T = 0.80, p = 0.185 with the geographic correction, p = 0.255 with the habitat correction, p = 0.180 with the phylogenetic correction), nor was it different when the risk transitioned from EN to CR species (T = −0.63, p = 0.415 with the geographic correction, p = 0.675 with the habitat correction, p = 0.760 with the phylogenetic correction). The results were similar when species with no identified threat were removed from the analysis: the average number of threat types per species increased when the level of extinction risk changed from LC to NT (T = 3.24, p = 0.005 with all corrections) and from NT to VU (T = 2.51, p = 0.005 with the geographic correction, p = 0.010 with the habitat correction, p = 0.020 with the phylogenetic correction); however, the number of threats was not different when the level of extinction risk changed from VU to EN (T = −0.16, p = 0.545 with the geographic correction, p = 0.575 with the habitat correction, p = 0.510 with the phylogenetic correction), nor when it changed from EN to CR species (T = −0.02, p = 0.120 with the geographic correction, p = 0.570 with the habitat correction, p = 0.495 with the phylogenetic correction). The orders exhibit different average numbers of threats per species, with some orders presenting particularly high values, including Sirenia, Proboscidae, and Perissodactyla (Table S4). The average number of threats per species within an order is strongly correlated with PDloss (Fig. 3; r = 0.55, t = 3.00, d.f. = 21, p = 0.007 with raw data; r = 0.58, t = 3.26, d.f. = 21, p = 0.004 with rank-transformed data; see also Fig. S1 for other models of species’ probabilities of extinction).

## Discussion

### Mammal Orders: there will be Winners and Losers

According to the IUCN50 model, we found that approximately 5% of extant mammalian phylogenetic diversity could be lost worldwide within the next 50 years. These predictions only consider worldwide extinctions; local extinctions are expected to be much higher. The proportion of mammal species at risk of extinction is high (25% of all mammals for which adequate data are available) and it could shortly be increased by the current 6% of NT species. In addition, according to the « Red List Index », which indicates the past rate at which species have shifted from one category to another on the Red List, mammal species whose extinction risk increases are more numerous than those whose risk decreases [40], [41].

It has been shown that if all species currently at risk of extinction (VU, EN, and CR species) were to go extinct, the corresponding loss of phylogenetic diversity would not be different from the expected extinction rate if species’ extinction risks were random [28]. Based on species’ extinction probabilities and modeling (IUCN50), within the next 50 years, the expected loss of phylogenetic diversity for all mammals is not different from what would be expected if mammalian extinction probabilities were unrelated to phylogeny, which indicates that even in a short time frame, the phylogenetic loss is expected to be random. The data-deficient species in the database could modify this result if these species were biased toward a high level of extinction risk. If the data-deficient species were all CR, the expected loss of phylogenetic diversity for all mammals would be higher than expected if mammalian extinction probabilities were unrelated to phylogenetic diversity.

It has been hypothesized that the existence of a phylogenetic signal in the extinction risk, with related species exhibiting similar extinction risks (as found in mammals, [11]), predicts higher losses of phylogenetic diversity than expected at random. However, a simulation-based study [42] demonstrated that phylogenetically clustered extinction risks are necessary, but not sufficient to predict an extensive loss of phylogenetic diversity. This might explain the apparent absence of an association between phylogeny and extinction risk, which can be observe based on the global amount of expected future mammalian phylogenetic diversity. The loss could be greater if the tree is heavily unbalanced, and the most distinct clades (with few descendents and relatives, sensu [32], [43]) are critically threatened [42]. The mammalian tree is heavily unbalanced due to Chiroptera and Rodentia, the most speciose orders, but we found that the most original species are not necessarily the most threatened, even when including data-deficient species classified as CR (see also [44] for Primates).

It is well known that similar levels of biodiversity can be obtained with very distinct species compositions. It was previously found that Primates are likely to lose more phylogenetic diversity than what is expected randomly, whereas the phylogenetic diversity loss in Carnivores is not different from what is expected randomly [11]. According to our results obtained using the model of extinction probabilities, IUCN50, and including all mammal orders (Table 2), at least Didelphimorphia, Chiroptera and Rodentia are likely to lose less phylogenetic diversity than what is expected randomly. Chiroptera and Rodentia represent 59% of the mammalian phylogenetic diversity that is not expected to decrease significantly in the near future. However, Diprotodontia, Cetartiodactyla, Primates, and Perissodactyla are likely to lose more phylogenetic diversity than what is expected randomly. Monotrema and Proboscidea are also likely to exhibit the most drastic declines in phylogenetic diversity because of their low numbers of species and the highly threatened status of several of their species. There will be "winners" and "losers" among the mammal orders. These results are different when data-deficient species are added to the analysis. When data-deficient species are classified as CR, certain orders (*e.g.*, Chiroptera and Rodentia) are re-equilibrated, with a more even balance observed between threatened and non-threatened species. Consequently, when data-deficient species are classified as CR, the winners disappear, while only two of the losing mammal orders are maintained as losers (Cetartiodactyla, Perissodactyla), and a new loser order is identified, most likely due to a high proportion of data-deficient species (Cingulata). Data-deficient species could modify estimations that predict lower future phylogenetic diversity for mammals than expected randomly if these species are particularly threatened, regardless of their phylogenetic position.

When species with sufficient data are analyzed, the most speciose orders, Chiroptera and Rodentia, could be winners, whereas several species-poor orders are among the losers. Based on similar observations, previous studies have hypothesized that the high levels of extinction risk in species-poor orders could be due to intrinsic inherited traits that may have resulted in the extinction of many of their members in the past. However, some studies have suggested that the cause of the species paucity of a particular order is unlikely to be the current vulnerability of the order to human-related threats because human-related threats are recent relative to the age of these orders [45]. In addition, although several traits have been suspected of increasing species’ extinction risks (Text S1 for a short review), geographical range size is the most important predictor of mammalian extinction risk, at both global and local levels, and most threatened mammals may have had their range sizes reduced, such that their current, small geographical ranges may reflect, but not explain a susceptibility to human impacts [8]. It is likely that the vulnerability of a whole order depends on the causes of the threats that affect its species within their habitats and geographical areas.

### Different Threats Affect Different Clades, Potentially Partially Attributed to where Species Live

Our analyses showed that three large phylogenetic clades differed in the types of threats that impact their species: Marsupiala and Monotremata species are primarily affected by exotic species and pathogens and, to a lesser extent, by climate change and pollution; Chiroptera species are primarily affected by human intrusions and disturbances, energy production and mining; and species from a sub-clade of Primates including Cebidae, Aotidae, Atelidae, Callitrichidae, Hominidae, Hylobatidae, Pitheciidae and Cercopithecidae are less affected by exotic species, climate change, and pollution, in contrast to the Marsupiala, but they are more affected by harvesting/hunting, urbanization, and agriculture.

We analyzed whether these associations between threats and phylogenetic clades could be due to specific spatial and habitat distributions. The main differences we found in terms of threat types were between the two hemispheres. Most orders are distributed between the two hemispheres and are therefore subjected to a variety of threats. However, among the Euarchontoglires, there are more Primate species in the Southern Hemisphere. Our analysis showed that species from a large sub-clade of Primates are primarily affected by major threats in the Southern Hemisphere, including urbanization, agriculture, and harvesting/hunting. The greater importance of urbanization and agriculture impacts in the Southern Hemisphere could be explained by the fact that many species affected by these factors have already been lost from the Northern Hemisphere (*e.g.*, [8]).

In the Northern Hemisphere, species are more affected by pollution, climate change, exotic species and pathogens, human disturbance, and the creation of transportation roads and railways. The threats common in Antarctica and Oceania are similar to the threats in the Northern Hemisphere. In Antarctica, climate change is a dominant threat. The trends found in Oceania might be due to human-driven impacts in Australia, including habitat alterations caused by the introduction of exotic organisms (*e.g.*, rabbits and foxes), changes in fire regimes, agriculture, sheep grazing, and urbanization that has fragmented ecosystems in both the arid interior and the more mesic coastal areas, all of which have resulted in many recent species extinctions [45], [46]. In Australia, the significant impact of threats, particularly invasive species, impacts the Marsupiala and, to a lesser extent, the Monotremata species, that live there. Recent anthropogenic impacts have been sufficiently profound in Australia to affect species on a continent-wide scale, regardless of their intrinsic biology [17].

We also observed differences in threats between the two hemispheres for marine species, which tend to be more affected by threats than terrestrial species [5]. In addition, terrestrial species dependent on freshwater were submitted to similar threats as marine species, predominantly threatened by climate change and pollution worldwide. However, marine species are polyphyletic and are distributed in three orders with relatives among terrestrial species, including all Sirenia species (freshwater is also a habitat for these species), a subset of Carnivora species, and a subset of Cetartiodactyla species. In addition, they are distributed in both the Northern and Southern Hemispheres. As a result, all of the orders are exposed to all types of threats.

For the Chiroptera, the dominant threats found are attributed to their habitat: underground sites such as caves and mines are critical to many Chiroptera worldwide [47]. Chiroptera are affected by human intrusion and disturbance, including speleology and mass visits by tourists [47], [48]. During hibernation, camera flashes and other disturbances provoke unnatural awakenings, which result in a decrease in energy and a risk that these organisms will not wake up in the spring [49]. Despite these threats, numerous Chiroptera species tend to exhibit low extinction risks, and as a result, the expected phylogenetic loss in this clade in the near future is low.

Our approach therefore identified a phylogenetic signal in the types of threat that affects large clades, including the Marsupiala, Chiroptera, and a subclade of Primates. However, even if geography and habitat explain differences in the types of threats affecting phylogenetic clades, they do not explain the levels of extinction risk for these clades. Indeed, Chiroptera are not particularly threatened. Among the Marsupiala, some orders are particularly threatened (*e.g.*, Diprotodontia), while others are not (*e.g.*, Didelphimorphia). A consequence of this finding is that biases in the threat types among mammalian lineages due to a particular spatial distribution or to an association with particular habitats, are not likely to translate into a higher extinction risk level.

### Threat Diversity Increases a Species’ Extinction Risk

Rather than the threat type, it is the accumulation of distinct threats for a single species that could be most responsible for its extinction risk and therefore explain the expected loss of phylogenetic diversity within each order. Some threats could be connected. For example, extensive use of pesticides in agriculture could lead to pollution, and transportation can introduce exotic, potentially invasive, species. Nevertheless, threats did not appear to be specifically correlated with the species they affect in our analyses, with the exception of agri- and aquaculture, urbanization, and harvesting/hunting. These three threat types have a greater impact in the Southern Hemisphere, which can explain the higher correlations among them. Additionally, the impact of transportation, which is correlated with urbanization and harvesting/hunting, can simply be explained by increased human density, a factor assumed to be a good general proxy for threat intensity [50].

Whether connected or not, accumulated threats can explain the extinction risk of a species. More precisely, we found that threat diversity increases from LC through NT to VU species. However, we found that, on average, the threat diversity is similar among VU, EN, and CR species. Threat diversity could determine the degradation of the status of a species from least-concerned (LC), through nearly threatened (NT) to at risk of extinction (VU+EN+CR). We used the number of major threats that affects a species as a broad estimate of threat diversity. A list of threats is a rough qualitative approximation of the level of impact within a species’ range. Some of the threats might be easier to identify than others. For instance, a natural disturbance factor might not be considered to represent a threat if a species is widespread. However, the same disturbance factor would be a threat if it affects the last populations of a species [22]. In addition, the updated threat classification is not necessarily exhaustive, and we only employed the first level of the threat classification system. Exploration of the second level of the threat classification system, which contains a more detailed definition of each threat, could be a promising direction for future studies. The third and last level of the threat classification system currently contains only illustrative examples of threats, rather than an actual classification system [22]. A clearer definition of this third level could improve the analysis of the causes of extinction risks. Moreover, integrating all of the contributing threats (direct and indirect), which is information that is presently unavailable for most taxa, could also improve the analysis of extinction risk because some threats (*e.g.*, hunting/harvesting) might be easier to treat through legislation, policy, and other conservation measures than other threats (climate change is typically driven by several contributing factors) [22].

In addition to how threats are classified by the IUCN Red List, the relationship between threat diversity and extinction risk could be impacted by the following factors: conservation actions, the area of distribution of each species and the threat intensity. Conservation actions tend to decrease the correlation between threat diversity and extinction risk by maintaining species associated with a high number of threats at an LC status. The most extreme example of this effect is *Lama guanicoe* (Cetartiodactyla). Although 10 major distinct threat types have been identified for this species according to the IUCN [6], it is classified as LC. This species is widespread and does not yet fulfill the criteria of extinction risk defined by the IUCN [23]. However, the sizes of its populations have drastically decreased, and their future depends on field conservation actions. Moreover, it has been shown that out of 181 mammal species whose status changed for genuine reasons (*i.e.* not just because of improved knowledge on the species) between 1996 and 2008, 37 species have exhibited a decreased extinction risk because of improvements in their abundance and distribution resulting from direct conservation interventions (Appendix 12 in [51]). Note that the status of some of these species has been improved since 2008: *e.g.*, *Gulo gulo*, of the Carnivora, was listed as VU in 1996, NT in 2008 and has had as status of LC since 2009; 5 threats have been identified for the status of this species, which depends on conservation actions. Because some threats (*e.g.*, hunting) might be easier to regulate than others (*e.g.*, climate change), and some threats might affect certain mammal lineages more than others, conservation actions may also modify the association between phylogeny and extinction risk. The geographic distribution of each species also impacts the relationship between threat diversity and extinction risk, as species with a wide geographic distribution can encounter many different threats because different geographic areas can be impacted by different threats. However, each population could be impacted by a low diversity of threats, and some populations might maintain a high abundance in less perturbed parts of the distribution range of their species, thus being classified as LC (*e.g.*, *Brachylagus idahoensis*, Lagomorpha), NT (*e.g.*, *Lutra lutra*, Carnivora) or VU (*e.g.*, *Hippocamelus antisensis*, Cetartiodactyla), depending on how well species are maintained in a specific area within their range. In contrast, species with very restricted areas of distribution can be affected by a highly stochastic fluctuation of their population sizes, thus being classified as CR (*e.g.*, *Peromyscus stephani*, Rodentia). For these species, a single threat can also be sufficient to critically threaten a species. For example, *Pipanacoctomys aureus* (Rodentia), which is known only from its type locality at Salar de Pipanaco in Catamarca province, Argentina, is classified as CR, and it is threatened by agricultural expansion [6]. Finally, for many species, it is only the occurrence of a threat, but not the threat intensity that is known. A single high-intensity threat could be more detrimental than several low-intensity threats. The impact of threat intensity on a species is also likely to depend on the size of a species’ distribution range, the threat extensiveness, and the existence of conservation actions.

Overall, the connection between threat diversity and extinction risk is an important factor in the search for the prevalence of intrinsic relative to extrinsic factors because patterns of extinction risk might reflect both the intrinsic sensitivity to threats and differences in threat intensity among regions [52]. One explanation for this connection between threat diversity and extinction risk is that some species exhibit traits that render them sensitive to several different threats, and we found a phylogenetic autocorrelation in the number of threats per species. A potential prevalence of intrinsic factors was more likely for factors that directly affect mammal species (*e.g.,* [10]). For example, hunted Artiodactyl species (order Cetartiodactyla) are more susceptible to extinction risks if they present lower reproduction rates. In contrast, non-hunted Artiodactyl species are more susceptible to extinction risks if they are found in less developed areas, where economic status affects the amount of money that a government can spend on conservation efforts and how its people exploit natural resources [14]. However, an accumulation of factors that directly affect mammal species could not explain the link between the number of threats and the extinction risk because only harvesting/hunting and pathogens affect mammal species directly. Other threat types affect species indirectly via impacts on their habitats. For example, urbanization, agriculture and aquaculture alter and reduce the favorable habitats available for certain species. In addition, transportation can fragment habitats.

Adding to the complexity of the mechanisms that contribute to a species’ extinction risk, the traits associated with sensitivity (or resistance) to a given threat can be very specific to the taxa considered, such as wing morphology in bats, and these traits might change from one order to another [53]. Better identification of important traits can be achieved by evaluating research based on the particular mammal order of interest [33], [52]. In addition, previous studies have demonstrated that the traits that make a species more vulnerable to extinction risks depend not only on their order, but also on the type of threat considered [13], [14]: certain traits can be associated with a single threat source, whereas others can be positively associated with one source and negatively with another. For instance, the Primates at risk of extinction due to hunting exhibit large body sizes; those at risk from logging show low ecological flexibility and larger geographic ranges; and those vulnerable to agriculture are likely to be arboreal and characterized by low-fruit diets [54]. When several threat types affect a single species, there is a chance that the species will exhibit at least one trait that makes it sensitive to one of the threats. However, if a species’ extinction risk increases with threat diversity and is due to trait-dependent and taxon-dependent traits, then species affected by a diversity of threats must accumulate detrimental traits and potentially disadvantageous genes if traits are inherited, making them sensitive to a variety of threat types, which is less likely.

Importance of intrinsic factors in addition to extrinsic factors was found only for large-bodied terrestrial mammals, with a limit of approximately 3 kg [55]. We found that Perissodactyla, Proboscidea, Cetartiodactyla, Primates, and Monotremata species are particularly affected, with between 3 and 5 threats observed per species on average, which impacts their future phylogenetic diversity. All Perissodactyla and Proboscidea, most Certartiodactyla, some Monotremata, and half of the Primate species have an adult body mass of greater than 3 kg [9]. Large body size is known to be associated with a variety of traits that could increase extinction risk. For example, large-bodied species are more tempting targets for hunting; they exhibit poor reproduction rates, resulting from a longer interval before reaching sexual maturity and smaller litters of larger offspring; and they are often specialists (see [52] for a review of potential causes of higher extinction rates among large-bodied species). A large body size could be associated with accumulation of detrimental traits and, hence, with sensitivity to a higher diversity of threats. This may at least partly explain the connection observed in this study between threat diversity and extinction risk. However, an increased risk of extinction for large-bodied species was found neither worldwide [8], [56], [57] nor in all taxa (*e.g.*, not in bats [53] and Artiodactyls [14]).

The trait-based explanation should therefore be completed with a geography- and habitat-based explanation: the increase in extinction risk associated with threat diversity could be due to an accumulation of human-driven impacts in some locations. Differences in the level of expected losses of phylogenetic diversity between orders could be primarily due to their localization in vulnerable geographical areas (*i.e.*, all marine areas and the Old World [5], [15]; see also Table S5) and habitats (*i.e.*, marine, artificial freshwater, wetlands, and forests, Table S6), but local data are needed to further test this hypothesis. Current efforts to define local red lists [58] are critical to precisely identify the local, regional, and global diversity of threats that could lead to species’ extinction [59]. According to our analysis, any action that reduces the diversity of threats that affect a species will have a significant impact on the future of mammalian phylogenetic diversity. There is a long-running debate in the conservation biology literature regarding whether we should protect geographic areas with many evolving lineages [60] or species-poor lineages whose loss would represent a loss of millions of years of past evolution [61]. An important point in this debate is that future radiations leading to the future phylogenetic tree of mammals are unpredictable [61], whereas extinctions might be predictable. Any extinct order is indefinitely removed from the future mammalian phylogenetic diversity.

In conclusion, human-driven causes of species extinction are now added to past and natural causes. Regardless of the taxa, geographical area and habitat considered, diversity among threats is detrimental to mammalian species diversity and phylogenetic diversity. Even if similar levels of worldwide mammalian phylogenetic diversity could be achieved with random species extinction risks, the phylogenetic clustering in the number of threats that affect each species could remove entire clades and seriously erode several orders, including Perissodactyla, Cetartiodactyla, Monotremata, Primates, and Proboscidea, potentially leading to major changes in the world composition of mammals with respect to their genotypes, phenotypes, and roles in ecosystems.

## Supporting Information

Figure S1 **Link between the average number of threats per species and the expected relative loss in phylogenetic diversity within each order.** (A) Isaac model and (B) Pessimistic model of species extinction risk (See Text S2 for a description of the models). The names of the orders impacted by the highest combined number of threats and the expected relative loss of phylogenetic diversity (PDloss) have been indicated.
(TIF)

Table S1 **Short description of the IUCN habitat classification.**
(DOC)

Table S2 **Test of the hypothesis H0 stating that observed loss in PD is not different from random loss (random species extinction probabilities).**
(DOC)

Table S3 **Results of the DPCoA analysis applied only to Primate species.**
(DOC)

Table S4 **Average number of threats affecting the species in each mammal order.**
(DOC)

Table S5 **Average number of threats affecting the species associated with each geographic area.**
(DOC)

Table S6 **Average number of threats affecting the species associated with each habitat.**
(DOC)

Text S1 **Traits associated with increasing extinction risk.**
(PDF)

Text S2 **Comparison of the IUCN50 model with two other models of species extinction probabilities.**
(PDF)

Text S3 **List of geographic areas.**
(PDF)

Text S4 **Details regarding the correlations among threats.**
(PDF)

Text S5 **Details regarding the correlations between the number of threats and the level of extinction risk.**
(PDF)
