# Supplementary figures and images for: Threat Diversity Will Erode Mammalian Phylogenetic Diversity in the Near Future
Source: PLoS One. 2012 Sep 28;7(9):e46235. doi: 10.1371/journal.pone.0046235 (PMC3460824; doi:10.1371/journal.pone.0046235)

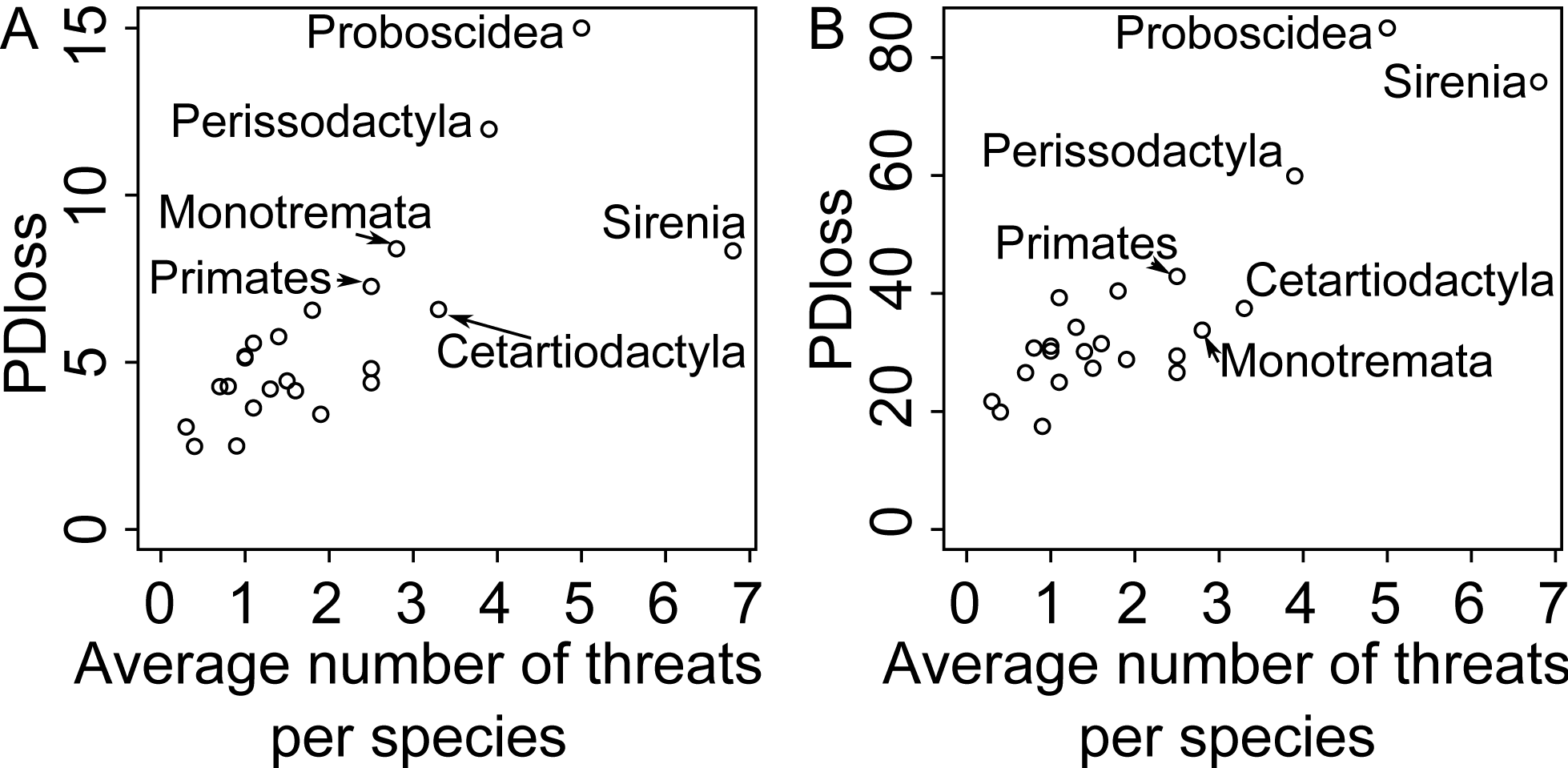

Supplement: Figure S1 — Link between the average number of threats per species and the expected relative loss in phylogenetic diversity within each order. (A) Isaac model and (B) Pessimistic model of species extinction risk (See Text S2 for a description of the models). The names of the orders impacted by the highest combined number of threats and the expected relative loss of phylogenetic diversity (PDloss) have been indicated. (TIF) [file pone.0046235.s001.tif]
