# Supplementary material for: Threat Diversity Will Erode Mammalian Phylogenetic Diversity in the Near Future
Source: PLoS One. 2012 Sep 28;7(9):e46235. doi: 10.1371/journal.pone.0046235 (PMC3460824; doi:10.1371/journal.pone.0046235)
Supplement: Table S1 — Short description of the IUCN habitat classification. (DOC) [file pone.0046235.s002.doc]

Table S1. Short description of the IUCN habitat classification

| **Habitats** | **Description and examples** |
| --- | --- |
| 1. Forest | Area with a high density of trees. Boreal, sub -arctic -antarctic, temperate, subtropical forests |
| 2. Savanna | Grassland ecosystem with scattered trees. Dry and moist savanna |
| 3. Shrubland | Vegetation dominated by shrubs. Boreal, sub -arctic -antarctic, temperate, subtropical, Mediterranean shrublands. |
| 4. Grassland | Vegetation dominated by grasses and other herbaceous plants. Tundra, sub -arctic -antarctic, temperate, subtropical grasslands. |
| 5. Wetlands (inland) | Area of ground that is saturated with water either permanently or seasonally. Rivers, bogs, permanent freshwater lakes… |
| 6. Rocky Areas | Mountain areas. Cliffs, inlands, mountain peaks… |
| 7. Caves | Natural opening in the ground. Including subterranean habitats |
| 8. Desert | Landscape that receives an extremely low amount of precipitation. Hot, temperate and cold desert. |
| 9. Marine Neritic | Shallow marine. Pelagic, subtidal areas, coral reef, seagrass, estuaries. |
| 10. Marine Oceanic | Deeper than marine neritic. Epipelagic, mesopelagic, bathypelagic, abyssopelagic areas. |
| 11. Marine Deep Benthic | Ecological region at the lowest level of a body of water, including the sediment surface and some sub-surface layers. Abyssal plains, seamount… |
| 12. Marine Intertidal | Area where land and sea met. Rocky shoreline, mud flats, tidepools. |
| 13. Marine Coastal/ Supratidal | Area that’s above high tide and which is regularly splashed but not submerged by the water. Coastal caves, coastal freshwater lakes… |
| 14. Artificial – Terrestrial | Terrestrial areas which are manipulated by humans. Arable land, pastureland, plantations, rural gardens, urban areas. |
| 15. Artificial – Aquatic | Aquatic areas manipulated by humans. Water storage area, ponds, salt exploitation sites, canals and drainage channels. |
| 16. Introduced Vegetation | - |
| 17. Other | - |
| 18. Unknown | - |

Based on IUCN (2011) IUCN Red List of Threatened Species. Version 2011.2. <[www.iucnredlist.org](http://www.iucnredlist.org/)>. Downloaded February 2012
