# Supplementary material for: Threat Diversity Will Erode Mammalian Phylogenetic Diversity in the Near Future
Source: PLoS One. 2012 Sep 28;7(9):e46235. doi: 10.1371/journal.pone.0046235 (PMC3460824; doi:10.1371/journal.pone.0046235)
Supplement: Table S2 — Test of the hypothesis H0 stating that observed loss in PD is not different from random loss (random species extinction probabilities). (DOC) [file pone.0046235.s003.doc]

**Table S2.** Test of the hypothesis H0 stating that observed loss in PD is not different from random loss (random species extinction probabilities).

(a) without data-deficient species (only species that have known phylogenetic positions were considered)

|  |  | IUCN 50 | | Isaac | | Pessimistic | |
| --- | --- | --- | --- | --- | --- | --- | --- |
| Orders | n | Relative loss (%) | p-value | Relative loss (%) | p-value | Relative loss (%) | p-value |
| Hyracoidea | 4 | 0.005 | 0.370 | 2.486 | 0.375 | 19.905 | 0.395 |
| Paucituberculata | 5 | 0.833 | 0.525 | 4.200 | 0.620 | 34.274 | 1.000 |
| Cingulata | 17 | 0.935 | 0.200 | 3.445 | 0.440 | 28.807 | 0.690 |
| **Didelphimorphia** | **68** | **1.763** | **0.020 (-)** | **3.065** | **0.020 (-)** | **21.699** | **0.015 (-)** |
| Pilosa | 9 | 2.517 | 0.840 | 2.497 | 0.840 | 17.483 | 0.815 |
| **Chiroptera** | **880** | **3.179** | **0.005 (-)** | **3.635** | **0.005 (-)** | **24.954** | **0.005 (-)** |
| Afrosoricida | 38 | 3.209 | 0.390 | 4.149 | 0.605 | 31.501 | 0.540 |
| **Sirenia** | 4 | 4.146 | 0.905 | 4.146 | 0.125 | **75.865** | **0.005(+)** |
| Macroscelidea | 12 | 4.271 | 0.870 | 8.224 | 0.980 | 30.762 | 0.600 |
| Carnivora | 252 | 4.444 | 0.395 | 4.386 | 0.945 | 29.406 | 0.090 |
| **Rodentia** | **1735** | **4.813** | **0.010 (-)** | **4.271** | **0.005 (-)** | **26.577** | **0.005 (-)** |
| Scandentia | 17 | 5.020 | 0.610 | 5.020 | 0.400 | 27.336 | 0.215 |
| Pholidota | 8 | 5.868 | 0.950 | 8.334 | 0.885 | 39.290 | 0.450 |
| Eulipotyphla | 330 | 6.658 | 0.720 | 5.130 | 0.940 | 31.067 | 0.850 |
| Dasyuromorphia | 61 | 6.819 | 0.710 | 5.175 | 0.785 | 30.239 | 0.825 |
| Lagomorpha | 81 | 6.956 | 0.510 | 4.811 | 0.595 | 26.605 | 0.905 |
| Peramelemorphia | 16 | 8.729 | 0.560 | 6.558 | 0.330 | 40.421 | 0.110 |
| **Diprotodontia** | **122** | **9.203** | **0.025 (+)** | **5.767** | **0.030 (+)** | 30.160 | 0.195 |
| **Cetartiodactyla** | **237** | **10.061** | **0.005 (+)** | **6.581** | **0.005 (+)** | **37.480** | **0.005 (+)** |
| **Primates** | **304** | **12.238** | **0.005 (+)** | **7.270** | **0.005 (+)** | **42.898** | **0.005 (+)** |
| Monotremata | 4 | 17.088 | 0.120 | 8.396 | 0.130 | 33.787 | 0.525 |
| Proboscidea | 2 | 23.500 | 0.115 | 15.000 | 0.115 | 85.000 | 0.055 |
| **Perissodactyla** | **14** | **25.502** | **0.005 (+)** | **11.972** | **0.005 (+)** | **59.919** | **0.005 (+)** |

The three orders with one only species in our data set were removed from the table. Tests obtained with the IUCN50 model are recalled here. The three orders with only one species in our dataset were removed from the table. The order Notoryctemorphia, with 2 DD species, was also discarded. *n* = number of species that are not classified as DD, and the number of DD species is given in brackets; relative loss = PDloss. Significance tests with α= 5% are highlighted in bold; a sign in brackets indicates whether PDloss is higher (+) or lower (-) than expected randomly. Orders are in the same order as in Table 2. Tests in red indicate differences between model Pessimistic and the two other models.

(b) assuming data-deficient species to be LC (only species that have known phylogenetic positions were considered)

|  |  | IUCN 50 | | Isaac | | Pessimistic | |
| --- | --- | --- | --- | --- | --- | --- | --- |
| Orders | n | Relative loss (%) | p-value | Relative loss (%) | p-value | Relative loss (%) | p-value |
| Hyracoidea | 4(0) | 0.005 | 0.165 | 2.486 | 0.180 | 19.905 | 0.155 |
| Paucituberculata | 5(0) | 0.833 | 0.605 | 4.200 | 0.825 | 34.274 | 0.805 |
| Cingulata | 17(3) | 6.412 | 0.220 | 2.610 | 0.530 | 22.220 | 0.805 |
| **Didelphimorphia** | **68(14)** | **1.405** | **0.030 (-)** | **2.837** | **0.025 (-)** | **20.544** | **0.020 (-)** |
| Pilosa | 9(0) | 2.517 | 0.905 | 2.497 | 0.990 | 17.483 | 1.000 |
| **Chiroptera** | **880(153)** | **2.611** | **0.005 (-)** | **3.327** | **0.005 (-)** | **23.359** | **0.005 (-)** |
| Afrosoricida | 38(4) | 2.963 | 0.405 | 3.949 | 0.740 | 30.112 | 0.400 |
| **Sirenia** | **4(0)** | 4.146 | 0.915 | 8.334 | 0.160 | **75.865** | **0.010 (+)** |
| Macroscelidea | 12(3) | 3.991 | 0.945 | 4.055 | 0.925 | 29.307 | 0.435 |
| **Carnivora** | **252(19)** | 4.147 | 0.700 | 4.237 | 0.690 | **28.559** | **0.010 (+)** |
| **Rodentia** | **1735(290)** | **4.101** | **0.010 (-)** | **3.935** | **0.005 (-)** | **24.930** | **0.005 (-)** |
| Scandentia | 17(3) | 4.607 | 0.785 | 4.081 | 0.580 | 25.412 | 0.320 |
| Pholidota | 8(0) | 5.868 | 0.925 | 5.573 | 0.730 | 39.290 | 0.270 |
| Eulipotyphla | 330(61) | 5.845 | 0.685 | 4.769 | 0.995 | 29.379 | 0.875 |
| Dasyuromorphia | 61(1) | 6.662 | 0.460 | 5.088 | 0.470 | 29.545 | 0.590 |
| Lagomorpha | 81(5) | 6.632 | 0.255 | 4.684 | 0.360 | 25.928 | 0.670 |
| Peramelemorphia | 16(2) | 7.889 | 0.485 | 6.871 | 0.245 | 38.450 | 0.045 |
| **Diprotodontia** | **122(2)** | **9.127** | **0.010 (+)** | **5.738** | **0.010 (+)** | **20.544** | **0.040 (+)** |
| **Cetartiodactyla** | **237(53)** | **7.436** | **0.035 (+)** | **5.391** | **0.020 (+)** | **32.025** | **0.005 (+)** |
| **Primates** | **304(15)** | **11.188** | **0.005 (+)** | **6.871** | **0.005 (+)** | **40.190** | **0.005 (+)** |
| Monotremata | 4(0) | 17.088 | 0.090 | 8.396 | 0.090 | 33.787 | 0.475 |
| Proboscidea | 2(0) | 23.500 | 0.095 | 15.000 | 0.070 | 85.000 | 0.010 |
| **Perissodactyla** | **14(0)** | **25.502** | **0.005 (+)** | **11.972** | **0.005 (+)** | **59.919** | **0.005 (+)** |

The three orders with one only species in our data set were removed from the table. Tests obtained with the IUCN50 model are recalled here. The three orders with only one species in our dataset were removed from the table. The order Notoryctemorphia, with 2 DD species, was also discarded. *n* = number of species that are not classified as DD, and the number of DD species is given in brackets; relative loss = PDloss. Significance tests with α= 5% are highlighted in bold; a sign in brackets indicates whether PDloss is higher (+) or lower (-) than expected randomly. Orders are in the same order as in Table 2. Tests in red indicate differences between model Pessimistic and the two other models.

(c) assuming data-deficient species to be CR (only species that have known phylogenetic positions were considered)

|  |  | IUCN 50 | | Isaac | | Pessimistic | |
| --- | --- | --- | --- | --- | --- | --- | --- |
| Orders | n | Relative loss (%) | p-value | Relative loss (%) | p-value | Relative loss (%) | p-value |
| Hyracoidea | 4(0) | 0.005 | 0.180 | 2.486 | 0.110 | 19.905 | 0.070 |
| Paucituberculata | 5(0) | 0.833 | 0.375 | 4.200 | 0.395 | 34.274 | 0.540 |
| **Cingulata** | **17(3)** | **23.536** | **0.035 (+)** | 8.518 | 0.275 | **43.388** | **0.045 (+)** |
| Didelphimorphia | 68(14) | 18.024 | 0.470 | 9.129 | 0.605 | 34.901 | 0.550 |
| Pilosa | 9(0) | 2.517 | 0.465 | 2.497 | 0.460 | 17.483 | 0.480 |
| **Chiroptera** | **880(153)** | 13.162 | 0.240 | 7.388 | 0.305 | **32.788** | **0.030 (-)** |
| Afrosoricida | 38(4) | 10.404 | 0.370 | 6.854 | 0.550 | 36.672 | 0.720 |
| **Sirenia** | **4(0)** | 4.146 | 0.575 | 8.334 | 0.935 | **75.865** | **0.035 (+)** |
| Macroscelidea | 12(3) | 10.321 | 0.770 | 6.292 | 0.745 | 35.219 | 0.770 |
| Carnivora | 252(19) | 10.488 | 0.145 | 6.692 | 0.315 | 33.946 | 0.730 |
| Rodentia | 1735(290) | 16.358 | 0.150 | 8.602 | 0.475 | 35.641 | 0.055 |
| Scandentia | 17(3) | 12.600 | 0.540 | 7.249 | 0.465 | 33.225 | 0.255 |
| Pholidota | 8(0) | 5.868 | 0.410 | 5.573 | 0.500 | 39.290 | 1.000 |
| Eulipotyphla | 330(61) | 17.699 | 0.260 | 9.365 | 0.270 | 39.365 | 0.440 |
| Dasyuromorphia | 61(1) | 8.435 | 0.070 | 5.774 | 0.135 | 30.992 | 0.255 |
| Lagomorpha | 81(5) | 8.518 | 0.260 | 5.423 | 0.330 | 27.687 | 0.360 |
| Peramelemorphia | 16(2) | 17.225 | 0.800 | 9.777 | 0.620 | 46.060 | 0.205 |
| Diprotodontia | 122(2) | 9.935 | 0.200 | 6.051 | 0.250 | 30.733 | 0.370 |
| **Cetartiodactyla** | **237(53)** | **29.747** | **0.005 (+)** | **13.733** | **0.005 (+)** | **51.281** | **0.005 (+)** |
| **Primates** | **304(15)** | 15.925 | 0.080 | **8.697** | **0.030 (+)** | **45.147** | **0.005 (+)** |
| Monotremata | 4(0) | 17.088 | 0.950 | 8.396 | 0.900 | 33.787 | 1.000 |
| Proboscidea | 2(0) | 23.500 | 0.870 | 15.000 | 0.910 | 85.000 | 0.085 |
| **Perissodactyla** | **14(0)** | **25.502** | **0.035 (+)** | **11.972** | **0.020 (+)** | **59.919** | **0.010 (+)** |

The three orders with one only species in our data set were removed from the table. Tests obtained with the IUCN50 model are recalled here The three orders with only one species in our dataset were removed from the table. The order Notoryctemorphia, with 2 DD species, was also discarded. *n* = number of species that are not classified as DD, and the number of DD species is given in brackets; relative loss = PDloss. Significance tests with α= 5% are highlighted in bold; a sign in brackets indicates whether PDloss is higher (+) or lower (-) than expected randomly. Orders are in the same order as in Table 2. Tests in red indicate differences between model IUCN50 and the two other models.
