# Supplementary material for: Threat Diversity Will Erode Mammalian Phylogenetic Diversity in the Near Future
Source: PLoS One. 2012 Sep 28;7(9):e46235. doi: 10.1371/journal.pone.0046235 (PMC3460824; doi:10.1371/journal.pone.0046235)
Supplement: Table S3 — Results of the DPCoA analysis applied only to Primate species. (DOC) [file pone.0046235.s004.doc]

**Table S3.** Results of the DPCoA analysis applied only to Primate species.

(a)

| **Families** | **Average coordinate of the species on the first axis** |
| --- | --- |
| First clade | |
| Lemuridae | -121.883 |
| Indriidae | -117.719 |
| Daubentoniidae | -114.416 |
| Lepilemuridae | -113.936 |
| Cheirogaleidae | -111.833 |
| Galagidae | -79.210 |
| Lorisidae | -79.132 |
| Tarsiidae | -69.479 |
| Second clade | |
| Hylobatidae | 15.925 |
| Hominidae | 16.664 |
| Cercopithecidae | 17.471 |
| Pitheciidae | 36.078 |
| Aotidae | 36.227 |
| Callitrichidae | 36.538 |
| Cebidae | 36.571 |
| Atelidae | 36.933 |

The first axis expressed 68% of the phylogenetic differences between the species affected by different types of threats. Higher absolute value means higher relative association with the corresponding threats (see b). Association measures are constrained by the phylogenetic relatedness among species. Families are used here to simplify the interpretation of the results but the mathematical approach is based on species and their phylogenetic relatedness.

(b)

| **Threat** | **Coordinate** |
| --- | --- |
| Threats affecting the first clade | |
| Pollution | -46.089 |
| Ecosystem change | -27.407 |
| Energy production | -18.076 |
| Agri- & aquaculture | -3.0420 |
| Hunting/harvesting | -1.951 |
| Threats affecting the second clade | |
| Exotics & pathogens | 0.543 |
| Urbanization | 21.520 |
| Intrusion | 22.179 |
| Transportation | 22.268 |

The effects of geological events and climate changes were not included in this analysis as they concerned only one species each for which we had phylogenetic information. Higher absolute value means higher relative association with the clade.
