# Supplementary material for: Threat Diversity Will Erode Mammalian Phylogenetic Diversity in the Near Future
Source: PLoS One. 2012 Sep 28;7(9):e46235. doi: 10.1371/journal.pone.0046235 (PMC3460824; doi:10.1371/journal.pone.0046235)
Supplement: Table S4 — Average number of threats affecting the species in each mammal order. (DOC) [file pone.0046235.s005.doc]

**Table S4.** Average number of threats affecting the species in each mammal order.

| **Order** | **Number of species** | **Mean of threats** |
| --- | --- | --- |
| Tubulidentata | 1 | 0 |
| Didelphimorphia | 68 | 0.25 |
| Hyracoidea | 4 | 0.25 |
| Rodentia | 1735 | 0.74 |
| Macroscelidea | 12 | 0.75 |
| Pilosa | 9 | 0.77 |
| Eulipotyphla | 330 | 1.02 |
| Chiroptera | 880 | 1.04 |
| Dasyuromorphia | 61 | 1.10 |
| Pholidota | 8 | 1.13 |
| Diprotodontia | 122 | 1.28 |
| Scandentia | 17 | 1.47 |
| Afrosoricida | 38 | 1.55 |
| Paucituberculata | 5 | 1.60 |
| Peramelemorpha | 16 | 1.81 |
| Cingulata | 17 | 1.88 |
| Dermoptera | 1 | 2 .00 |
| Microbiotheria | 1 | 2.00 |
| Lagomorpha | 81 | 2.49 |
| Primates | 304 | 2.49 |
| Carnivora | 252 | 2.52 |
| Monotremata | 4 | 2.72 |
| Cetartiodactyla | 237 | 3.22 |
| Perissodactyla | 14 | 3.86 |
| Proboscidea | 2 | 5.00 |
| Sirenia | 4 | 6.75 |
| Mammals | 4223 | 1.28 |

Only mammals with phylogenetic position were kept in our analyses. The number of species in our data set is indicated. See Text S5 for details.
