# Supplementary material for: Threat Diversity Will Erode Mammalian Phylogenetic Diversity in the Near Future
Source: PLoS One. 2012 Sep 28;7(9):e46235. doi: 10.1371/journal.pone.0046235 (PMC3460824; doi:10.1371/journal.pone.0046235)
Supplement: Table S5 — Average number of threats affecting the species associated with each geographic area. (DOC) [file pone.0046235.s006.doc]

**Table S5.** Average number of threats affecting the species associated with each geographic area.

| **Geographic area** | **Number of species** | **Mean of threats worldwide** |
| --- | --- | --- |
| Mesoamerica | 561 | 1.03 |
| South America | 908 | 1.12 |
| Caribbean Islands | 169 | 1.12 |
| SubSaharan Africa | 993 | 1.19 |
| Oceania | 494 | 1.23 |
| North America | 421 | 1.41 |
| East Asia | 560 | 1.41 |
| West-central Asia | 411 | 1.49 |
| North Africa | 166 | 1.60 |
| South southeast Asia | 1005 | 1.73 |
| North Asia | 286 | 1.74 |
| Europe | 213 | 1.91 |
| Antarctic | 17 | 2.12 |
| Pacific Antarctic | 12 | 2.25 |
| Indian Ocean Antarctic | 15 | 2.27 |
| Atlantic Antarctic | 13 | 2.38 |
| Southeast Pacific | 25 | 2.48 |
| Eastern central Pacific | 30 | 2.77 |
| Southwest Atlantic | 27 | 2.78 |
| Southwest Pacific | 23 | 2.83 |
| Northeast Pacific | 24 | 2.88 |
| Southeast Atlantic | 22 | 3.00 |
| Northwest Pacific | 33 | 3.12 |
| Eastern Indian Ocean | 25 | 3.16 |
| Western Indian Ocean | 21 | 3.24 |
| West-central Atlantic | 20 | 3 .25 |
| Northeast Atlantic | 22 | 3.27 |
| Eastern-central Atlantic | 21 | 3.33 |
| Western-central Pacific | 20 | 3.35 |
| Northwest Atlantic | 26 | 3.46 |
| Mediterranean Black sea | 10 | 3.50 |
| Arctic Sea | 12 | 4.00 |

The species list has been divided into geographic areas. The number of species in our data set is indicated. See Text S4 for details
