# Supplementary material for: Threat Diversity Will Erode Mammalian Phylogenetic Diversity in the Near Future
Source: PLoS One. 2012 Sep 28;7(9):e46235. doi: 10.1371/journal.pone.0046235 (PMC3460824; doi:10.1371/journal.pone.0046235)
Supplement: Table S6 — Average number of threats affecting the species associated with each habitat. (DOC) [file pone.0046235.s007.doc]

**Table S6.** Average number of threats affecting the species associated with each habitat.

| **Habitat** | **Number of species** | **Mean of threats worldwide** |
| --- | --- | --- |
| Unknown habitat | 41 | 0.80 |
| Artificial Terrestrial | 1362 | 0.92 |
| Rocky areas | 409 | 1.03 |
| Savanna | 715 | 1.08 |
| Desert | 391 | 1.15 |
| Shrubland | 1317 | 1.20 |
| Caves | 415 | 1.22 |
| Other habitats | 16 | 1.25 |
| Grassland | 1152 | 1.30 |
| Forest | 3055 | 1.34 |
| Wetlands | 388 | 1.78 |
| Artificial Aquatic and Marine | 53 | 2.79 |
| Marine Coastal Supratidal | 84 | 2.89 |
| Marine Oceanic | 61 | 3.03 |
| Introduced vegetation | 19 | 3.32 |
| Marine Intertidal | 59 | 3.42 |
| Marine Neritic | 85 | 3.54 |

The species list has been divided into habitats. The number of species in our data set is indicated. See Text S4 for details
