# Supplementary material for: Threat Diversity Will Erode Mammalian Phylogenetic Diversity in the Near Future
Source: PLoS One. 2012 Sep 28;7(9):e46235. doi: 10.1371/journal.pone.0046235 (PMC3460824; doi:10.1371/journal.pone.0046235)
Supplement: Text S1 — Traits associated with increasing extinction risk. (PDF) [file pone.0046235.s008.pdf]

Text S1 Traits associated with increasing extinction risk

Studies that searched for potential traits associated with species sensitivity to threats have considered extinction risk as defined by recent or ongoing population decline only (category A1 of the IUCN data base = recent decline in population size at a specified level). Identified traits for mammals were:

- lower population growth (faster population growth would lead to faster recovery from disturbance which reduces population size, [1]; slow life histories reduce ability of species to compensate for increased mortality and have been connected to overexploitation, [2,3]; slow-breeding animals tend to be enable to recover from population crashes following high rates of predation [4]);
- long generation times (which might decrease the rate at which species can adapt to change, [5]);
- large home range (higher individual energetic requirement, lower population density, vulnerability to reserve edge effects, [1] and references therein);
- high trophic level ([2], bioaccumulation effect: organisms that occupy the highest trophic level are more susceptible to pollution);
- low dispersal ability ([5]);
- habitat and diet specialism [6];
- large body size (globally, the life-history traits that increase species' vulnerability to anthropogenic threats scale with body size, see e.g. [7]; large-bodied species are longer to reach sexual maturity, they have smaller litters of larger offspring and larger individual home ranges, they are often specialists and also more tempting targets for hunters).

Other additional traits were also invoked such as attractiveness to people as pets ([8] and references therein). Among the frequently discussed traits, behavioural and physiological traits have often been neglected. Within mammals, lower risk of extinction and reduced exposure of environmental stress is observed with "the sleep or hide behaviour" (including hibernation, topor, use of burrows) associated with a related suite of physiological adaptations allowing mammals to function at lower metabolic rates and/or buffer them from changing physical elements [1,8]. Other identified traits might be considered as non-heritable characteristics only indirectly associated with species phylogeny such as:

- small geographic range size (narrow distribution, an effect believed to be intensified by habitat loss [3]; species with larger geographic distribution spread the risk among populations residing at different spatial locations);
- low abundances (Allee effect, [9]; individuals interact in order to ensure their own survival or reproduction, the absence of congeners produce a negative effect on their fitness by reducing the rate limiting mating opportunities to meet; for mammals, it has been shown that monogamous species, and species where males have to defend small harems were more prone to vulnerability, [10]);
- isolated populations (demographic and environmental stochasticity in isolated areas might lead to natural extinctions, [10]; island populations, generally isolated, are more prone to extinction than mainland populations, with island endemic species having the highest extinction rates, [11]).

References

1. Cardillo, M (2003) Biological determinants of extinction risk: why are smaller species less vulnerable? Anim Conserv 6: 63-69.

2. Purvis, A, Gittleman JL, Cowlishaw G, Mace GM (2000) Predicting extinction risk in declining species. P R Soc B-Biol Sci 267: 1947-1952.

3. Fritz, SA, Bininda-Emonds ORP, Purvis A (2009) Geographical variation in predictors of mammalian extinction risk: big is bad, but only in the tropics. Ecol Lett 12: 538-549.

4. Fisher, DO, Blomberg SP, Owens IPF (2003) Extrinsic versus intrinsic factors in the decline and extinction of Australian marsupials. P R Soc B-Biol Sci 270: 1801-1808.

5. Jones, KE, Purvis A, Gittleman JL (2003) Biological correlates of extinction risk in bats. Am Nat 161: 601-614.

6. Boyles, JG, Storm JJ (2007) The perils of picky eating: dietary breadth is related to extinction risk in insectivorous bats. PloS ONE 7: e672.

7. Fritz, SA, Purvis A (2010) Phylogenetic diversity does not capture body size variation at risk in the world's mammals. P R Soc B-Biol Sci 277: 2435-2441.

8. Liow, LH, Fortelius M, Lintulaakso K, Mannila H, Stenseth NC (2009) Lower extinction risk in sleep-or-hide mammals. Am Nat 173: 264-272.

9. Bessa-Gomes, C, Legendre S, Clobert J (2004) Allee effects, mating systems and the extinction risk in populations with two sexes. Ecol Lett 7: 802-812.

10. Brashares, JS (2003) Ecological, behavioral, and life-history correlates of mammal extinctions in West Africa. Conserv Biol 17: 733-743.

11. Francham, R (1998) Inbreeding and extinction: island populations. Conserv Biol 12: 665-675.
