# Supplementary material for: Threat Diversity Will Erode Mammalian Phylogenetic Diversity in the Near Future
Source: PLoS One. 2012 Sep 28;7(9):e46235. doi: 10.1371/journal.pone.0046235 (PMC3460824; doi:10.1371/journal.pone.0046235)
Supplement: Text S2 — Comparison of the IUCN50 model with two other models of species extinction probabilities. (PDF) [file pone.0046235.s009.pdf]

Text S2 Comparison of the IUCN50 model with two other models of species extinction probabilities

Extinction probabilities for model IUCN50 are: LC = 0.00005, NT = 0.004, VU = 0.05, EN = 0.42 and CR = 0.97 in 50 years.

Two other models of species extinction probabilities defined by [1] were analysed:

- The model Isaac was inferred from [2] using the IUCN extinction probability designation for the category VU. Extinction probabilities are: LC = 0.025, NT = 0.05, VU = 0.1, EN = 0.2 and CR = 0.4 in 100 years. The model Isaac is more optimistic than model IUCN50 (at least for the most threatened categories).
- The model Pessimistic is based on arbitrary extinction probabilities: LC = 0.2, NT = 0.4, VU = 0.8, EN = 0.9 and CR = 0.99, without time frame. The model IUCN50 is the least arbitrary.

With these two models, the scenarios of expected loss in mammal phylogenetic diversity (PDloss) were as follows:

With the pessimistic scenario and considering only mammals for which adequate data are available (predicted loss = 28.5%, average random loss = 28.4%, p = 0.65), the predicted loss of mammal phylogenetic diversity was not quantitatively different from the loss expected if species extinction risks were independent of their phylogeny. When adding data-deficient species as LC, the predicted mammal phylogenetic diversity loss (26.7%) is also not quantitatively different from the loss expected if species' extinction risks were independent of their phylogeny (average random loss=26.7%, p=0.945). In contrast, when adding data-deficient species as CR, the predicted mammal phylogenetic diversity loss (36.4%) is quantitatively higher than the loss expected if species' extinction risks were independent of their phylogeny (average random loss=35.3%, p=0.005).

With the more optimistic scenario (model Isaac) and considering only mammals for which adequate data are available, the observed loss was significantly different from that expected randomly but lower (observed loss = 4.6%, average random loss = 4.7%, p = 0.030). When adding data-deficient species as LC, the predicted mammal phylogenetic diversity loss (4.2%) is also not quantitatively different from the loss expected if species' extinction risks were independent of their phylogeny (average random loss=4.3%, p=0.055). In contrast, when adding data-deficient species as CR, the predicted mammal phylogenetic diversity loss (8.3%) is quantitatively higher than the loss expected if species' extinction risks were independent of their phylogeny (average random loss=8.0%, p=0.005).

As for the IUCN50 model, whatever the scenario of species extinction probabilities, some orders could lose more PD than others (Table S2).

Regarding the correlation between average number of threats per species and expected PDloss, the results obtained with the models Isaac and Pessimistic confirm those obtained with IUCN50:

The average number of threats per species of an order is strongly correlated with PDloss (Fig. S2) ($r = 0.76$, $t=5.37$, $p<10^{-4}$ with model Isaac; $r = 0.87$, $t=8.18$, $p<10^{-7}$ with model Pessimistic).

The model Pessimistic slightly differ from the other models by estimating high expected PD loss for the Sirenia (Table S2), which is in agreement with the very high number of threats per species (Fig. S1). All Sirenia species are Vulnerable, and this category is given high probability of extinction with model Pessimistic.

References

1. Mooers AØ, Faith DP, Maddison WP (2008) Converting endangered species categories to probabilities of extinction for phylogenetic conservation prioritization. PloS ONE 3:e3700.

2. Isaac NJB, Turvey ST, Collen B, Waterman C, Baillie JEM (2007) Mammals on the EDGE: conservation priorities based on threat and phylogeny. PloS ONE 2:e296.
