# Supplementary material for: Threat Diversity Will Erode Mammalian Phylogenetic Diversity in the Near Future
Source: PLoS One. 2012 Sep 28;7(9):e46235. doi: 10.1371/journal.pone.0046235 (PMC3460824; doi:10.1371/journal.pone.0046235)
Supplement: Text S3 — List of geographic areas. (PDF) [file pone.0046235.s010.pdf]

Text S3 List of geographic areas

Land regions

Antarctic
Caribbean Islands
East Asia
Europe
Mesoamerica
North Africa
North America
North Asia
Oceania
South America
South & Southeast Asia
Sub-Saharan Africa
West & Central Asia

FAO Marine Areas

Arctic Sea
Atlantic – Antarctic
Atlantic – eastern central
Atlantic – northeast
Atlantic – northwest
Atlantic – southeast
Atlantic – southwest
Atlantic – western central
Indian Ocean – Antarctic
Indian Ocean – eastern
Indian Ocean – western
Mediterranean and Black Sea
Pacific – Antarctic
Pacific – eastern central
Pacific – northeast
Pacific – northwest
Pacific – southeast
Pacific – southwest
Pacific – western central
