# Supplementary material for: Threat Diversity Will Erode Mammalian Phylogenetic Diversity in the Near Future
Source: PLoS One. 2012 Sep 28;7(9):e46235. doi: 10.1371/journal.pone.0046235 (PMC3460824; doi:10.1371/journal.pone.0046235)
Supplement: Text S4 — Details regarding the correlations among threats. (PDF) [file pone.0046235.s011.pdf]

Text S4 Details regarding the correlations among threats

Pair-wise Phi correlations between threats:

	Urbanization	Agri- & aquaculture	Energy production	Transportation	Harvesting / Hunting	Intrusion	Ecosystem changes	Exotics & pathogens	Pollution	Geological events
Agri- & aquaculture	**0.431**									
Energy production	0.174	0.142								
Transportation	**0.333**	0.237	0.161							
Harvesting / Hunting	**0.355**	**0.582**	0.164	**0.251**						
Intrusion	0.221	0.090	0.241	0.148	0.152					
Ecosystem changes	0.172	**0.301**	0.111	0.211	0.217	0.128				
Exotics & pathogens	0.187	0.198	0.066	0.249	0.229	0.172	0.250			
Pollution	0.123	0.083	0.071	0.194	0.163	0.172	0.161	0.231		
Geological events	0.065	0.023	0.039	0.024	0.021	0.043	0.009	0.047	0.021	
Climate change	0.095	0.093	0.023	0.157	0.120	0.128	0.136	0.241	0.164	0.192

We have checked with the mammal data that randomizing the values of each of threat vectors led to a correlation between the averaged randomized phi coefficient and the number of species concerned by the two compared threats close to zero (with 1000 randomizations, we obtained a correlation between the average randomized phi coefficient and the number of species concerned by the two compared threats equal to 0.041). The low phi coefficients obtained between Geological events and the other threats are thus true lacks of association.

All correlations were found to be lower than 0.25 except:

ϕ(Agri- & Aquaculture, Harvesting/Hunting) = 0.58
ϕ(Urbanization, Agri- & Aquaculture) = 0.43
ϕ(Urbanization, Harvesting/Hunting) = 0.36
ϕ(Urbanization, Transportation) = 0.33
ϕ(Agri- & Aquaculture, Ecosystem changes) = 0.30
ϕ(Transportation, Harvesting/Hunting) = 0.25
