# Supplementary material for: Threat Diversity Will Erode Mammalian Phylogenetic Diversity in the Near Future
Source: PLoS One. 2012 Sep 28;7(9):e46235. doi: 10.1371/journal.pone.0046235 (PMC3460824; doi:10.1371/journal.pone.0046235)
Supplement: Text S5 — Details regarding the correlations between the number of threats and the level of extinction risk. (PDF) [file pone.0046235.s012.pdf]

Text S5 Details regarding the correlations between the number of threats and the level of extinction risk

Moran's test

To check the validity of the correlation test between the rank-transformed number of threats and the rank-transformed level of extinction risk of mammal species, we used the residuals of the linear model of the level of extinction risk explained by the number of threats (rank-transformed data). We have analysed the phylogeny-, geography- and habitat-based autocorrelation in the residuals by Moran's test [1]. Moran's test requires a matrix of proximities between species. For phylogeny-based autocorrelation, we measured the proximity between two species as the sum of branch-length between their first common ancestor and the root of the mammal phylogeny. For geography-based autocorrelation, we first established a table with species as rows, geographic areas as columns and 0/1 as entries depending on the occurrence of each species within each geographic area. We then calculated the geographic proximity between two species with Dice index [2]. We used the same approach for habitat-based autocorrelation, by establishing a table with species as rows, habitats as columns and 0/1 as entries depending on the occurrence of each species within each habitat.

Lapointe & Garland's permutation approach

We then used permutation tests based on [3] to correct p-values in the test of correlation between the rank-transformed number of threats and the rank-transformed level of extinction risk of mammal species based on phylogenetic-, geography- and habitat-based null models. These tests are based on distances among species. Phylogenetic distances were calculated as the sum of branch lengths on the smallest path that connects to species on the phylogenetic tree. Geography-based distances and habitat-based distances were calculated as 1-Dice index.

Average numbers of threats per species

We also calculated the average number of threats per order (Table S4), the average number of threats affecting species that occur in each geographic area (Table S5) and each habitat (Table S6). Geographic and habitat means are given for information. However, the number of threats affecting a species is worldwide. We do not know whether the species is affected by all threats within each of its location or whether threats are different from one location to another. As a result these means should be interpreted as "species occurring within this geographic area (or habitat) at least in part of their range tend to have few or many threats worldwide".

References

1. Moran PAP (1948) The interpretation of statistical maps. J R Stat Soc Ser B-Stat Methodol 10: 243-251.

2. Dice LR (1945) Measures of the amount of ecologic association between species. Ecology 26:297-302.

3. Lapointe FJ, Garland T (2001) A generalized permutation model for the analysis of cross-species data. J Classif 18: 109-127.
